# Tumor Genomics, Metastatic Patterns, and Prognosis in Leiomyosarcoma: A Single-Center Retrospective Cohort Study

**DOI:** 10.3390/cancers17213544

**Published:** 2025-11-01

**Authors:** Hayes Pearce, Yu-Cherng Chang, Sarah Wishnek Metalonis, Brandon Edward Rose, Emily E. Jonczak, Ty Subhawong, Gina D’Amato, Julie Grossman, Patricia Castillo, Marilyn Huang, Marco Magistri, Francis Hornicek, Andrew E. Rosenberg, Jonathan C. Trent, Francesco Alessandrino

**Affiliations:** 1 Leonard M. Miller School of Medicine, University of Miami, Miami, FL 33136, USA; whp20@med.miami.edu; 2 Department of Radiology, Jackson Memorial Hospital, Miami, FL 33136, USA; ychang40@med.miami.edu; 3 Division of Biostatistics, Department of Public Health, University of Miami, Miami, FL 33136, USA; smetalonis@med.miami.edu; 4 Department of Medicine, University of Miami, Miami, FL 33136, USA; brose@med.miami.edu; 5 Division of Medical Oncology, Department of Medicine, University of Miami, Miami, FL 33136, USA; emily.jonczak@med.miami.edu (E.E.J.); gina.damato@med.miami.edu (G.D.); jtrent@med.miami.edu (J.C.T.); 6 Sylvester Comprehensive Cancer Center, Miami, FL 33136, USA; tsubhawong@med.miami.edu (T.S.); juliegrossman@med.miami.edu (J.G.); rcastillo3@med.miami.edu (P.C.); 7 Department of Radiology, University of Miami, Miami, FL 33136, USA; 8 Division of Surgical Oncology, Department of Surgery, University of Miami, Miami, FL 33136, USA; 9 Division of Gynecologic Oncology, UVA Health, Charlottesville, VA 22903, USA; msh8f@uvahealth.org; 10 Caris Life Sciences, 4610 South 44th Place, Phoenix, AZ 85040, USA; mmagistri@carisls.org; 11 Department of Orthopedic Surgery, University of Miami, Miami, FL 33136, USA; fjh21@med.miami.edu; 12 Department of Pathology & Laboratory Medicine, University of Miami, Miami, FL 33136, USA; arosenberg@med.miami.edu

**Keywords:** leiomyosarcoma, soft tissue sarcoma, biomarkers, genomic profiling, next-generation sequencing, metastatic patterns, precision oncology

## Abstract

**Simple Summary:**

Leiomyosarcoma (LMS) is a rare and aggressive soft tissue sarcoma that can originate from uterine and non-uterine sites. Current understanding of the prognostic implications of genomic alterations and metastatic patterns is limited. The aim of our retrospective study was to assess the prevalence of common mutations, patterns of metastatic spread, and their associations with survival in 110 LMS patients who underwent both genomic testing and longitudinal follow-up at our institution. We found that metastatic patterns differed between uterine and non-uterine LMS, and that *ATRX* mutations and pleural metastases were independently associated with worse overall survival. These findings highlight the prognostic relevance of integrating genomic and metastatic features and may inform risk stratification and therapeutic decision-making in LMS.

**Abstract:**

Background/Objectives: The prognostic associations of tumor genomics and metastatic patterns remain incompletely defined in leiomyosarcoma (LMS). We investigated the association between tumor mutations, sites of metastasis, and survival in patients with LMS. Methods: This single-center retrospective cohort study evaluated 110 patients with biopsy-proven LMS who underwent genomic testing between January 2009 and May 2023. Associations between tumor mutations, metastatic sites, and uterine vs. non-uterine LMS were assessed using χ^2^ or Fisher’s exact test. Progression-free survival/recurrence-free survival (PFS/RFS) and overall survival (OS) were estimated with the Kaplan–Meier method and compared using the log-rank test, and subsequent Cox proportional hazards regression examined associations of OS and PFS/RFS with tumor mutations and metastatic sites. Results: The study included 110 subjects (F/M: 81/29; median age, 57 years; 25/110 with metastatic disease). Overall, the most common mutations were in *TP53* (74/110, 67%) and *RB1* (24/110, 22%), and the most common metastatic sites were the lungs (79/99, 80%) and liver (37/99, 37%). In terms of metastatic patterns, peritoneal (24/50, 48%), pelvic (23/50, 46%), and pleural (9/50, 18%) metastases were more common in the uLMS group (*p* = 0.001, 0.01, and 0.04, respectively), whereas liver (27/60, 45%) and retroperitoneal (15/60, 25%) metastases were more common in the nuLMS group (*p* = 0.03 and 0.04, respectively). *ATRX* mutations (17/110, 15%) and pleural metastases (11/99, 11%) were independently associated with lower OS. Predictive survival models were generated, demonstrating variable interdependent associations between genomic alterations, metastatic sites, and outcomes (OS and PFS/RFS). Post hoc analysis of an independent cohort (N = 2606) demonstrated that *ATRX* mutations were similarly associated with lower OS (28.95 vs. 33.86 months; *p* = 0.006). Conclusions: Our study identifies differences in metastatic patterns between uterine and non-uterine LMS and highlights the adverse prognostic association of *ATRX* mutations and pleural metastases in a leiomyosarcoma-specific cohort.

## 1. Introduction

Leiomyosarcoma (LMS) is an aggressive, rare malignant neoplasm of the smooth muscle that accounts for 10–20% of all soft tissue sarcomas [1]. It occurs most commonly in the uterus [2], though it can originate from other anatomic sites, including the retroperitoneum, extremity soft tissues, hollow and solid viscera, and vasculature. Uterine LMS (uLMS) represents 1–3% of all uterine malignancies [3,4]. Despite relatively frequent early diagnosis, the five-year survival rate for primary uLMS varies from 25 to 76%, dropping to 10–15% when metastatic [4]. For non-uterine LMS (nuLMS), estimates for five-year overall survival range from 30% to 70% [5]. The heterogeneity of LMS likely contributes to the wide range of survival estimates. The current mainstay of treatment is a combination of surgery, chemotherapy, and radiation, with no FDA-approved targeted therapies [6]. Key genomic features of LMS include frequent inactivation of *TP53* and *RB1* and whole-genome duplications [7,8,9].

Numerous studies have identified how mutational status and metastatic patterns inform prognosis in other uterine malignancies, such as uterine serous carcinoma [10] and endometrial carcinoma [11], as well as in sarcomas, such as gastrointestinal stromal tumors [12]. Lung metastases have been identified as an independent predictor of poor prognosis in both uLMS and nuLMS [13]. Given the emergence of precision oncology, mutational status has become central to predicting prognosis and treatment response. Favorable prognostic biomarkers have been identified in early-stage uterine LMS, such as ER/PR positivity [14]. However, owing to its heterogeneity and high mortality rate, the overall prognostic role of biomarkers in LMS remains poorly understood. Despite advances in molecular profiling, no validated biomarkers have been incorporated into standard-of-care clinical practice to stratify risk, predict survival, or predict treatment response [15].

Substantially expanding upon our preliminary data [16], the objective of this study was to investigate differences in the mutational status and metastatic patterns between uLMS and nuLMS, and to evaluate whether genomic mutations were associated with metastatic patterns and whether these, alone or in combination, correlated with progression-free survival (PFS), recurrence-free survival (RFS), overall survival (OS), and/or demographics.

## 2. Materials and Methods

### 2.1. Patient Population

This institutional review board (IRB)-approved, Health Insurance Portability and Accountability Act (HIPAA)-compliant retrospective study was conducted at a single institution and included patients with histopathologically confirmed LMS, diagnosed by a sarcoma pathologist (A.R.), who underwent molecular testing between January 2009 and May 2023. Electronic health records were queried for subjects who met the following criteria: (1) age > 18 years at the time of histopathologic diagnosis, (2) mention of “leiomyosarcoma” on histopathology analysis, and (3) availability of next-generation sequencing (NGS) data, yielding a total of 210 subjects. Subjects were excluded if they had (1) non-leiomyosarcoma histology (n = 96), (2) NGS performed for unrelated conditions (n = 2), or (3) insufficient tissue submitted for NGS analysis (n = 2). Application of these exclusion criteria resulted in a final cohort of 110 patients.

### 2.2. Clinical and Histopathologic Data

Medical records were reviewed to extract clinical and histopathological data. The collected variables included demographics, date of initial diagnosis, tumor stage and grade, genomic testing results, type of LMS (uterine vs. non-uterine), initial treatment modality (systemic therapy, radiotherapy, or surgery), date and sites of metastatic and recurrent disease, type of imaging studies performed from diagnosis to last follow-up, treatment for recurrent disease, progression or recurrence date, and date of death or last follow-up.

### 2.3. Imaging Review

Computed tomography (CT) of the chest, abdomen, and pelvis; fluorodeoxyglucose F 18 (FDG) positron emission tomography (PET)/CT; and magnetic resonance imaging (MRI) studies performed from diagnosis to the last available follow-up were reviewed by a radiologist-in-training (Y.C.) and confirmed by a diagnostic radiologist with more than 5 years of experience interpreting sarcoma imaging studies (F.A.). The date of the first imaging scan showing metastasis or recurrence was recorded along with the sites, as described in previous studies [10,11]. Lymph nodes were considered involved when they had a short-axis diameter ≥ 1.5 cm or when they showed unequivocal increase (>20% increase) in short axis on follow-up imaging or by histopathology. Any new lesion identified on follow-up imaging after curative treatment was defined as recurrence based on pathologic confirmation or if it showed unequivocal growth on follow-up imaging, defined as a >20% increase in the sum of diameters compared to baseline or nadir (with an absolute increase of at least 5 mm) according to RECIST 1.1 [17]. Any lesion present before curative treatment was considered metastatic, based on pathologic confirmation or if it showed unequivocal growth on follow-up imaging, according to RECIST 1.1 [17].

### 2.4. Genomic Testing

Genomic testing was performed using NGS on either the primary tissue at diagnosis or the tissue obtained at recurrence. Molecular testing was performed using either Caris Life Sciences (Phoenix, AZ, USA) or FoundationOne CDx (Foundation Medicine Inc., Cambridge, MA, USA) genomic profiling assays. Details of the specific NGS testing are available from Caris Life Sciences or FoundationOne CDx. Among the genes covered, we focused our analyses on the 12 most frequently mutated genes in this cohort.

### 2.5. Statistical Analysis

Associations between mutational status, location of metastases or recurrence, type of LMS, ethnicity, and race were assessed using Chi-square or Fisher’s exact tests when 20% or more of the cells had expected counts less than 5. The FDR method with the MULTTEST procedure was used to control the false discovery rate (Type I errors) for all resultant *p*-values from Fisher’s exact or chi-square association tests [18]. Statistical significance was set at *p* < 0.05. OS was measured in months from the date of the initial diagnosis to death from any cause and censored at the date of the last follow-up in live patients. Kaplan–Meier survival curves were generated, and univariate Cox proportional hazards regression was used to examine the association of mutational status or sites of recurrence/metastases with OS or PFS/RFS. OS and PFS/RFS curves were computed for all subjects. Statistical significance was determined using the log-rank test (statistical significance at *p* < 0.05).

Cox proportional hazards regression models for OS and PFS/RFS were performed using a backward stepwise regression approach, beginning with a fully saturated model, incorporating variables that had a log-rank *p*-value of less than 0.02 from the Kaplan–Meier analysis and were then gradually reduced by convergence using the PHREG procedure. Likelihood ratio tests were used to test model predictability and significance for additional individuals and/or the collection of variables. Statistical computations were performed using SAS software (version 9.4).

### 2.6. Post Hoc Survival Analysis of ATRX Mutations

Following initial statistical analysis, we further examined the association between the presence of *ATRX* mutations in leiomyosarcoma and overall survival. A Kaplan-Meier survival curve of an independent cohort of 2606 subjects with LMS in the Caris Life Sciences’ Comprehensive Oncology Data Explorer (CODEai) platform was constructed, including 561 subjects with *ATRX* mutations detected by NGS.

## 3. Results

### 3.1. Patient Characteristics

**Figure 1:**
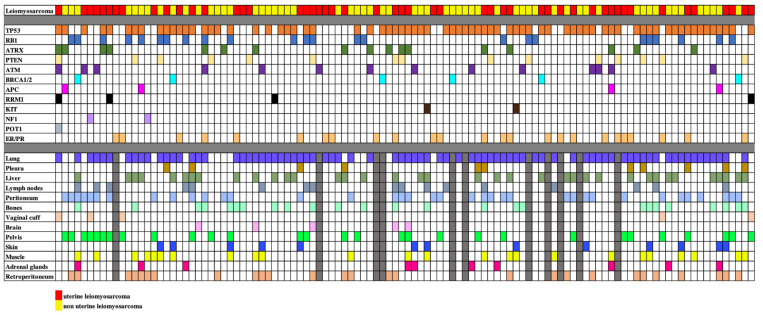
Cell plot showing the mutational status and sites of metastatic disease of our study sample of 110 patients with uterine and non-uterine leiomyosarcomas. Rows illustrate the leiomyosarcoma types, the different mutations and the different metastatic sites, each identified by a different color. Each column represents a single patient. When a mutation and metastasis are present, the relevant box is filled. When data is not available, boxes are filled in gray.

A total of 110 patients with LMS were included in the final analysis. Fifty subjects had uLMS (45.5%) and 60 subjects had nuLMS (54.5%). 51 (46%) patients presented with localized disease, 34 (31%) with locally advanced disease, and 25 (23%) with metastatic disease at diagnosis (Table 1). Median OS and PFS/RFS were 47 months (IQR: 30–63.5 months), and 11 months (IQR: 5–17.5) for localized LMS, 37 months (IQR: 19–57 months) and 9 months (IQR: 4–21 months) for locally advanced LMS, and 27 months (IQR: 15–38 months) and 9 months (IQR: 3–12.5 months) for metastatic disease respectively. Within the cohort, 79 (71.8%) had high-grade disease, 22 (20.0%) had intermediate-grade disease, 4 (3.6%) had low-grade disease, and 5 (4.5%) had unknown grade. The clinical, genomic, and metastatic phenotypes of the participants are summarized in Table 1. A cell plot of the cohort is provided in Figure 1.

### 3.2. Molecular Aberrations

Targeted sequencing of the tumor was performed from surgical specimens in 72 subjects (65%) and biopsies in 38 (35%). The most frequently mutated genes detected by NGS were *TP53* (74/110; 67%), *RB1* (24/110; 22%), *ATRX* (17/110; 15%), *PTEN* (15/110; 14%), and *ATM* (14/110; 13%) (Table 1). ER/PR expression was present in 25/110 patients (23%).

### 3.3. Location of Metastases

**Figure 2:**
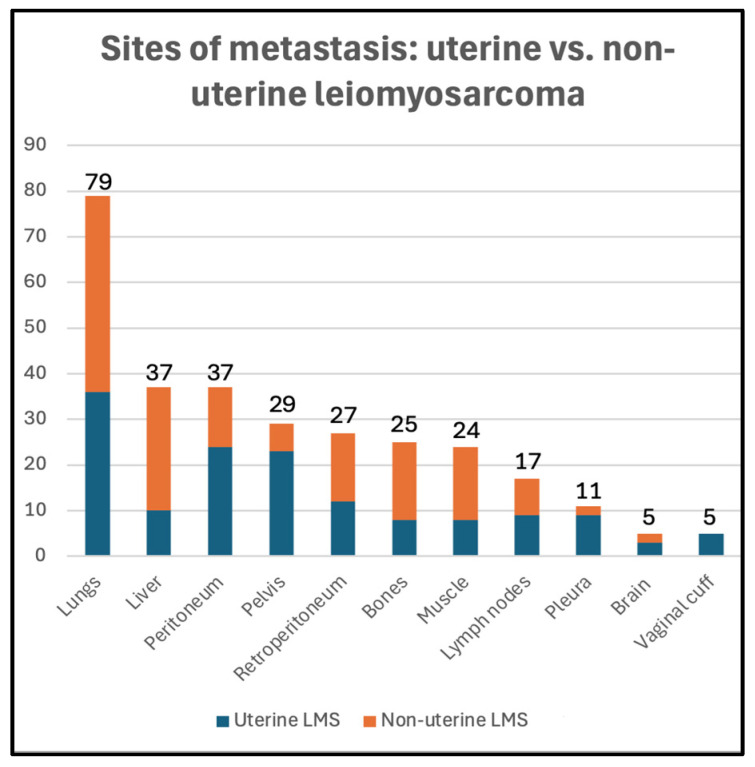
Metastatic sites in uterine (blue) vs. non-uterine (orange) leiomyosarcoma in our study sample.

Twenty-five patients had evidence of metastasis at the time of diagnosis (23%), 74 patients developed metastasis during follow-up (67%), and 11 patients did not show evidence of metastasis during follow-up (10%) (Table 1). The distribution of metastatic sites among uterine and non-uterine LMS is summarized in Figure 2. The lungs were the most common site of metastasis (79/99, 80%), followed by the peritoneum (37/99, 37%), liver (37/99, 37%), pelvis (29/99, 29%), and retroperitoneum (27/99, 27%).

### 3.4. Associations Between LMS Types, Sites of Metastases, and Mutational Status

Peritoneal (24/50, 48%), pelvic (23/50, 46%), and pleural (9/50, 18%) metastases were more common in the uLMS group (*p* = 0.001, 0.01, and 0.04, respectively). Vaginal cuff recurrence was identified only in the uLMS group (5/44, 11%) (*p* = 0.04). Liver (27/60, 45%) and retroperitoneal (15/60, 25%) metastases were more common in the nuLMS group (*p* = 0.03, 0.04, respectively).

ER/PR expression was identified only in the uLMS group (25/50, 50%) (*p* = 0.001). After correction for the false discovery rate, no other statistically significant association between molecular aberrations and the type of LMS was identified.

Adrenal gland metastases were more common in subjects with *APC* mutations (*APC* mutations 3/4 vs. no *APC* mutations 7/95; *p* = 0.03). After correcting for the false discovery rate, no other statistically significant association between molecular aberrations and sites of metastases was identified.

### 3.5. Association of Mutational Status with Survival Endpoints

**Figure 3:**
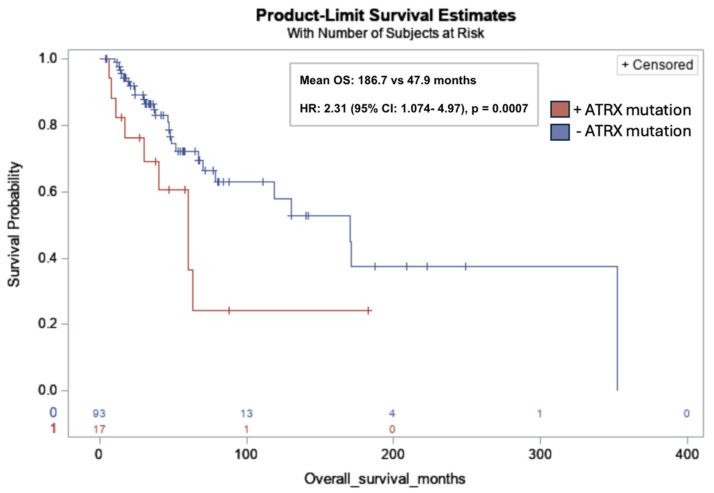
Kaplan–Meier overall survival curve for leiomyosarcoma patients with *ATRX* mutations (red) versus without *ATRX* mutations (blue). *ATRX* mutation was significantly associated with lower OS in all subjects (17/110, 15%) (mean OS: 186.7 months vs. 47.9 months; HR: 2.31; 95% CI: 1.074, 4.97; *p* = 0.0007). Risk table at bottom of graph: 0: *ATRX* mutation (red); 1: No *ATRX* mutation (blue).

*ATRX* mutations were significantly associated with lower OS in all subjects (mean OS: 186.7 months vs. 47.9 months; HR: 2.31; 95% CI: 1.074, 4.97; *p* = 0.001) (Figure 3).

*BRCA1/2* mutations were significantly associated with lower PFS/RFS in non-Hispanic subjects (3/83, 4%) (mean OS: 69 months vs. 2.5 months; HR: 19.38; 95% CI: 3.7, 100.91; *p* = 0.004).

No other significant associations were observed between molecular aberrations and OS or PFS/RFS, including no association between ER/PR expression and OS or PFS/RFS in all patients with uLMS (*p* = 0.42 and 0.31, respectively).

### 3.6. Association of Metastases with Survival Endpoints

**Figure 4:**
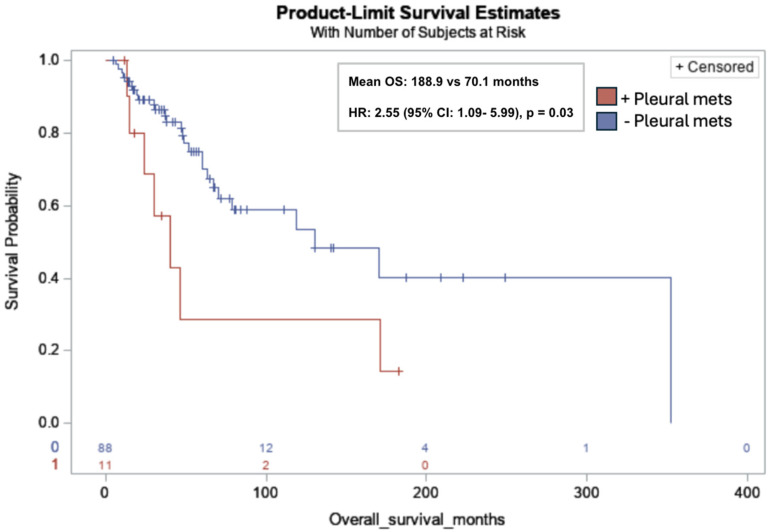
Kaplan-Meier overall survival curve for leiomyosarcoma patients with pleural metastases (red) versus without pleural metastases (blue). Pleural metastases (pleural mets) were significantly associated with lower OS in all subjects (11/99, 11%) (mean OS: 188.9 months vs. 70.1 months; Hazard Ratio (HR): 2.55; 95% CI: 1.09, 5.99; *p* = 0.03). Risk table at bottom of graph: 0: no pleural metastases (red); 1: pleural metastases (blue).

Pleural metastases were significantly associated with lower OS in all subjects (mean OS: 188.9 months vs. 70.1 months; HR: 2.55; 95% CI: 1.09, 5.99; *p* = 0.03) (Figure 4). No additional associations between the sites of metastasis and survival endpoints were identified.

### 3.7. Predictive Survival Models

The Cox proportional hazards regression model for OS began with a full, 14-variable model (*ATRX*, *KIT*, *APC*, and *RRM1* mutations; pleural, lung, bones, peritoneum, vaginal cuff, pelvis and liver metastases; race, ethnicity and sex). After reducing the full OS model based on the Wald test statistics for individual variables and likelihood ratio tests (−2 Log Likelihood), the final reduced model was determined with five parameters (pleural metastases, *KIT* mutations, *RRM1* mutations, bone metastases, and *APC* mutations), N = 96. Of the parameters included in the final model, pleural metastases, *KIT* and *RRM1* mutations were found to predict OS (pleural metastases: *p* = 0.01, HR: 3.15; CI: 1.28, 7.73; *KIT* mutation, *p* = 0.02; HR: 12.88; CI: 1.38, 120.2; *RRM1* mutation: *p* = 0.02, HR: 4.28; CI: 1.23, 14.86).

Similarly, the Cox proportional hazards regression model for PFS/RFS began with a 14-variable model (*BRCA1* and 2, *ATRX*, *KIT*, and *RRM1* mutations, brain, bones, lungs, pleura, peritoneum, liver, and vaginal cuff metastases; race, ethnicity, and sex), all with log-rank *p*-values < 0.20. After reducing the model 10 times based on the Wald test statistics for individual variables and likelihood ratio tests (−2 Log Likelihood), a final PFS/RFS reduced model was determined with four parameters (*BRCA1/2* mutations, *ATRX* mutation, bone metastases, and *KIT* mutations), N = 93. The four parameters included in the model were found to predict PFS/RFS (*BRCA1/2* mutations: *p* = 0.007, HR: 9.38, CI: 1.83, 48.04; *ATRX* mutation: *p* = 0.04; HR: 2.33, CI: 1.04, 5.2; bone metastases: *p* = 0.03; HR: 0.31, CI: 0.1, 0.89; *KIT* mutation, *p* = 0.02; HR: 13.65; CI: 1.41, 132.1).

### 3.8. Post Hoc Survival Analysis of ATRX Mutations in LMS

**Figure 5:**
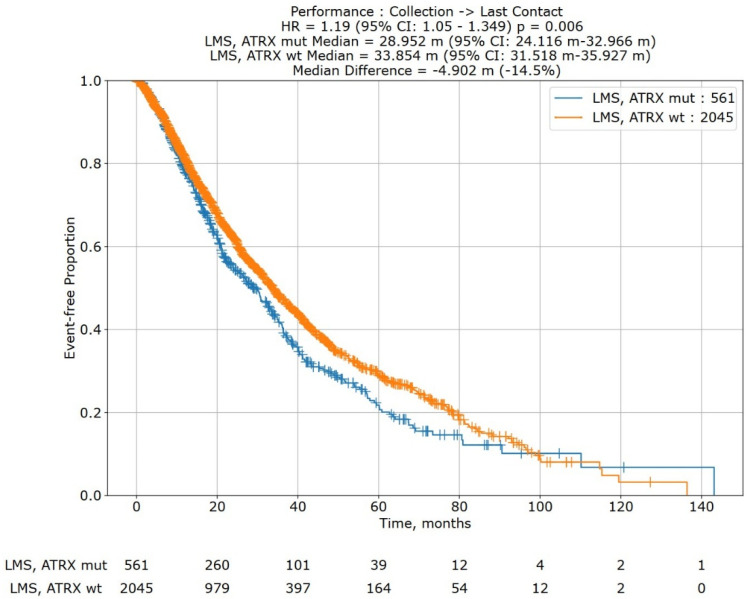
Kaplan–Meier overall survival curve for leiomyosarcoma patients with *ATRX* mutations (blue; cohort 1) versus leiomyosarcoma patients without *ATRX* mutations (orange; cohort 2). Survival analysis is of data extracted from Caris Life Sciences’ Comprehensive Oncology Data Explorer (CODEai) platform (N = 2606). *ATRX* mutations were significantly associated with decreased overall survival (28.952 months vs. 33.858 months; HR: 1.19, 95% CI: 1.05, 1.349; *p* = 0.006). A risk table is at the bottom of the graph.

In a Kaplan–Meier survival analysis of 2606 subjects from the Caris Life Sciences’ Comprehensive Oncology Data Explorer (CODEai) platform (Figure 5), *ATRX* mutations (N = 561) were significantly associated with decreased overall survival (median OS: 28.952 months vs. 33.854 months; HR: 1.19; 95% CI: 1.05, 1.349; *p* = 0.006).

## 4. Discussion

Understanding the prognostic significance of genomic alterations and metastatic patterns in LMS is essential to improving outcomes and facilitating appropriate prognostic discussions with patients. We identified certain mutations and metastatic sites that have implications for survival outcomes.

*ATRX* mutations, present in 15.5% of the cohort, were significantly associated with decreased OS, with a mean OS of 47.9 months compared to 186.7 months in patients without detected mutations (HR = 2.31; 95% CI: 1.07–4.97; *p* = 0.001). *ATRX* is a chromatin remodeler and tumor suppressor gene that plays an important role in numerous cell regulatory pathways related to DNA replication and transcriptional regulation [19]. To provide context, loss-of-function mutations in *ATRX* are common in sarcomas. A genetic analysis of a large cohort of 2138 sarcomas spanning over 45 sarcoma subtypes identified >10% loss of *ATRX* expression across various soft tissue sarcoma subtypes, with uLMS having the highest mutation rate of up to one in three cases [20]. In vitro and in vivo studies by Darmusey et al. demonstrate that *ATRX*-mutant sarcoma tissue exhibits immune downregulation, specifically through reduced mast cell recruitment. This diminished immune response was associated with accelerated tumor growth, suggesting a mechanism by which *ATRX* loss facilitates immune escape and drives tumor progression [21].

Our study reinforces *ATRX* mutations as a marker of poor prognosis in leiomyosarcoma, aligning with prior observations in other sarcoma subtypes and extending this association to both uterine and non-uterine disease in a leiomyosarcoma-specific cohort [22,23,24]. The prognostic potential of *ATRX* mutations in LMS is further supported by our post hoc analysis of data extracted from Caris Life Sciences’ Comprehensive Oncology Data Explorer (CODEai) platform. Elimusertib, an inhibitor of ATR kinase, has shown an increase in overall survival in vivo in mouse models with ATRX-mutant uterine LMS [25]. In the United States, a phase I/II trial for elimusertib in refractory solid tumors is underway [26]. Notably, loss of *ATRX* expression has been identified as a poor prognostic marker in other malignancies, such as glioblastoma multiforme and low-grade gliomas [27,28].

In our prognostic model, other mutations were associated with decreased survival: *RRM1* mutations were associated with lower OS, and *KIT* mutations were associated with lower OS and PFS/RFS in both uLMS and nuLMS. Research on the prognostic role of *RRM1* in LMS is limited; however, a 44-subject retrospective study of LMS failed to identify a relationship between *RRM1* expression and survival [29]. While *KIT* is often mutated in gastrointestinal stromal tumors (GIST), which represents a targetable mutation with clinical benefit, it is rare in LMS, with mutations occurring in 1.8% of our cohort. Therapies targeting *KIT* have not shown significant clinical benefits in LMS [30].

We did not find a significant association between ER/PR expression and survival in patients with uLMS. This contrasts with several studies on FIGO stage I uLMS, including a study of 147 participants with FIGO stage I uLMS that demonstrated improved OS with higher PR expression, but failed to demonstrate improved survival in advanced-stage uLMS [31,32]. The lack of association in our study could be attributed to the relatively small number of patients with FIGO stage I uLMS in our cohort (28/50, 56%) [15].

*BRCA1/2* mutations were associated with a lower PFS/RFS in non-Hispanic subjects. Patients with *BRCA1/2*-mutant uLMS may benefit from PARP inhibitors. Seligson et al. identified a subset of 4 patients with uLMS who had lasting clinical benefits from PARP inhibitors, and multiple case reports have shown beneficial results [33,34,35].

In our cohort, the lungs were the most common site of metastasis, followed by the liver, peritoneum, and pelvis. While peritoneal, pleural, and pelvic metastases were significantly more common in the uLMS group, liver and retroperitoneal metastases were more common in the nuLMS group. Our findings concur with those of previous studies that investigated the metastatic pattern of uLMS, with minimal discrepancies between studies [36,37].

Pleural metastases occurred in 11% of our cohort and were significantly associated with lower OS in all subjects, with a mean OS of 70.1 months and an HR of 2.55 (95% CI: 1.09, 5.99; *p* = 0.03). Although prior studies have shown that lung metastases are an independent predictor of poor prognosis [13], to date, no studies have specifically identified the prognostic role of pleural metastases in uLMS and nuLMS. Mechanistically, as discussed by Gonnelli et al., protein-rich fluid in malignant pleural effusions contains oncogenic, angiogenic, and immunosuppressive factors such as VEGF and interleukin-10, providing a potential explanation for the association between pleural metastases and poor clinical outcomes [38]. Opportunities for further research include evaluating whether survival is influenced by the number of pleural metastatic foci and the potential benefit of cytoreductive surgery.

Surprisingly, bone metastases were linked to significantly improved PFS/RFS in our prognostic model, with an HR of 0.31 (95% CI: 0.1, 0.89, *p* = 0.03). In a retrospective study by LiBrizzi et al. of 396 patients with LMS, there was no significant association between bone metastases and OS [39]. However, a case series and systematic review by Contartese et al. found that spinal bone metastases were significantly associated with worse OS in uLMS [40]. Given the relative rarity and heterogeneity of bone metastases in LMS, further research is needed to clarify their prognostic impact on survival.

Several limitations of this study should be noted. This was a retrospective study with inherent selection bias, for which the relatively small sample size may have failed to show associations between types of LMS, molecular aberrations, and metastatic patterns, as well as the prognostic value of mutations and metastatic patterns. In our cohort, the majority of subjects (89.1%) underwent NGS after the initiation of systemic therapy and/or radiotherapy, which may confound interpretation of associations between genomic alterations and clinical outcomes. Additionally, variability in tumor grade and other histopathologic features, including the predominance of high-grade disease in our cohort (71.8%), may have influenced survival outcomes. Given the small sample size, we used proportional hazards regression and post hoc analysis of *ATRX*-mutant LMS to refine our assessment. Furthermore, we built a prognostic model for LMS, irrespective of uterine vs. non-uterine origin, which may have limited the prognostic value.

## 5. Conclusions

Understanding the clinical significance of the genomic landscape and metastatic patterns in LMS is essential for advancing care and improving outcomes. Our study demonstrates distinct differences in metastatic behavior between uterine and non-uterine LMS and highlights the adverse prognostic association of *ATRX* mutations and pleural metastases. Recognizing these indicators may help clinicians better assess disease severity and refine individualized treatment strategies. Future studies should validate these findings in larger, multi-institutional cohorts, continue to explore the biological mechanisms underlying *ATRX*-driven progression and pleural spread, and evaluate whether integrating genomic and metastatic features can refine prognostic models and inform biomarker-driven therapeutic approaches.

## Figures and Tables

**Table 1:** Clinical characteristics of a retrospective cohort of 110 patients with uterine or non-uterine leiomyosarcoma.

Characteristic	Number Total (110 Subjects)	Uterine LMS (N = 50)	Non-Uterine LMS (N = 60)
**Age at diagnosis (mean, standard deviation in years)**	57.65 ± 12.10	54.08 ± 8.33	60.62 ± 13.90
**Sex**			
**Female**	81 (73.6%)	50 (100%)	31 (51.7%)
**Male**	29 (26.4%)	0	29 (48.3%)
**Stage at diagnosis**			
**Localized**	51 (46%)	33 (66%) *	18 (30%)
**Locally advanced**	34 (31%)	8 (16%) **	26 (43%)
**Metastatic**	25 (23%)	9 (18%)	16 (27%)
**Race**			
**Black**	27 (24.8%)	15 (30%)	12 (20%)
**White**	79 (72.5%)	34 (68%)	45 (75%)
**Asian**	2 (1.8%)	1 (2%)	1 (1.7%)
**American Indian/Alaska Native**	1 (0.92%)	0	1 (1.7%)
**Ethnicity**			
**Hispanic**	27 (24.5%)	15 (30%)	12 (20%)
**Non-Hispanic**	83 (75.5%)	35 (70%)	48 (80%)
**Mutational status**			
**TP53**	74 (67.3%)	28 (56%)	46 (76.7%)
**RB1**	24 (21.8%)	7 (14%)	17 (28.3%)
**ATRX**	17 (15.5%)	11 (22%)	6 (10%)
**PTEN**	15 (13.6%)	9 (18%)	6 (10%)
**ATM**	14 (12.7%)	7 (14%)	7 (11.7%)
**BRCA1/2**	6 (5.5%)	1 (2%)	5 (8.3%)
**APC**	4 (3.6%)	1 (2%)	3 (5%)
**RRM1**	4 (3.6%)	3 (6%)	1 (1.7%)
**KIT**	2 (1.8%)	0	2 (3.3%)
**NF1**	2 (1.8%)	1 (2%)	1 (1.7%)
**POT1**	1 (0.9%)	1 (2%)	0
**ER/PR-positive**	25 (23%)	25 (50%)	0
**Metastatic sites**			
**Lungs**	79 (79.8%)	36 (72%)	43 (71.2%)
**Peritoneum**	37 (37.4%)	10 (20%)	27 (38.3%)
**Liver**	37 (37.4%)	24 (48%)	13 (21.7%)
**Pelvis**	29 (29.3%)	23 (46%)	6 (10%)
**Retroperitoneum**	27 (27.3%)	12 (24%)	15 (25%)
**Bones**	25 (25.3%)	8 (16%)	17 (28.3%)
**Muscle**	24 (24.2%)	8 (16%)	16 (26.7%)
**Lymph nodes**	17 (17.2%)	9 (18%)	8 (13.3%)
**Pleura**	11 (11.1%)	9 (18%)	2 (3.3%)
**Brain**	5 (5.1%)	3 (6%)	2 (3.3%)
**Vaginal cuff**	5 (5.1%)	5 (10%)	0

* FIGO stage I: 28 (56%); FIGO stage II: 1 (2%); localized with FIGO stage not provided: 4 (8%). ** FIGO stage III: 8 (16%); FIGO stage IVA: 0.

## Data Availability

Deidentified patient data may be requested by researchers with approved proposals by contacting the corresponding author at falessandrino@med.miami.edu.

## Abbreviations

The following abbreviations are used in this manuscript:

LMS	Leiomyosarcoma
uLMS	Uterine leiomyosarcoma
nuLMS	Non-uterine leiomyosarcoma
NGS	Next-generation sequencing
PFS	Progression-free survival
RFS	Recurrence-free survival
OS	Overall survival
HR	Hazard ratio
CI	Confidence interval
IRB	Institutional Review Board
HIPAA	Health Insurance Portability and Accountability Act
CT	Computed tomography
PET	Positron emission tomography
FDG	Fluorodeoxyglucose
MRI	Magnetic resonance imaging
RECIST	Response Evaluation Criteria in Solid Tumors
IQR	Interquartile range
FDA	Food and Drug Administration
CODEai	Comprehensive Oncology Data Explorer (Caris Life Sciences)
ER	Estrogen receptor
PR	Progesterone receptor
VEGF	Vascular endothelial growth factor
